# Evaluation of the Effect of PEGylated Single-Walled Carbon Nanotubes on Viability and Proliferation of Jurkat Cells

**Published:** 2012

**Authors:** Naghmeh Hadidi, Seyed Farshad Hosseini Shirazi, Farzad Kobarfard, Nastaran Nafissi-Varchehd, Reza Aboofazeli

**Affiliations:** a *Department of Pharmaceutics, School of Pharmacy, Shaheed Beheshti University of MedicalmSciences, Tehran, Iran.*; b *Department of Pharmacology & Toxicology, School of Pharmacy, Shaheed Beheshti University of Medical Sciences, Tehran, Iran.*; c *Department of Pharmaceutical Chemistry, School of Pharmacy, Shaheed Beheshti University of Medical Sciences, Tehran, Iran.*; d *Department of Pharmaceutical Biotechnology, School of Pharmacy, Shaheed Beheshti University of Medical Sciences, Tehran, Iran.*

**Keywords:** Phospholipid-PEG, Cytotoxicity, Flow cytometry, Functionalization, Cell culture

## Abstract

Among the numerous nanosized drug delivery systems currently under investigation, carbon nanotubes (CNTs), regardless of being single or multiple-walled, offer several advantages and are considered as promising candidates for drug targeting. Despite the valuable potentials of CNTs in drug delivery, their toxicity still remains an important issue. After the PEGylation of single-walled CNTs (SWCNTs) with phospholipid-PEG (Pl-PEG) conjugates to prepare water-dispersible nanostructures, the present study was designed to evaluate whether the functionalization with Pl-PEG derivatives could alter the cytotoxic response of cells in culture, affect their viability and proliferation. *In-vitro* cytotoxicity screens were performed on cultured Jurkat cells. The SWCNTs samples used in this exposure were pristine SWCNTs, Pl-PEG 2000/5000-SWCNTs at various concentrations. Jurkat cells were first incubated for 3 h at 37°C with test materials and seeded in 6-well culture plates at a given concentration. The plates were then incubated for 24, 48 and 72 h at 37°C in a 5% CO_2_ humidified incubator. Cell Viability and proliferation assay were performed using trypan blue exclusion test and the cell cycle kinetic status of Jurkat cells was analyzed by flow cytometry. Cell morphology was finally studied using double staining technique and a fluorescence microscope. We found that, regardless of the duration of exposure, functionalized SWCNTs were substantially less toxic, compared to pure SWCNTs and that the molecular weight of Pl-PEGs played an important role at higher concentrations. In conclusion, our noncovalent protocol seemed to be effective for increasing SWCNTs biocompatibility.

## Introduction

Nanotechnology, shortened to *nanotech*, is the study of controlling of matter on an atomic and molecular scale and generally deals with structures sized between 1 to 100 nanometer in at least one dimension. Nanotechnology has changed our lives dramatically and influenced every sector of the research and engineering. This technology has been used successfully in the design of particulate systems with uniform particle size, desired surface characteristics and geometrical forms and therefore, have led to the development of the new field of nanomedicine which includes many applications of nanomaterials and nanodevices for diagnostic and therapeutic purposes. From the pharmaceutical point of view, not only nanotechnology plays an important role in the production of novel drug delivery systems for controlled release and drug targeting, but also this field helps to improve the solubility and bioavailability of poorly soluble drugs (1-5).

Among the nanopharmaceuticals currently being used or under investigation, one can mention carbon nanotubes (CNTs). CNTs are carbon-based nanomaterials and considered as hollow cylinders in nanoscale dimensions, with diameters in the range of 0.5-100 nm and lengths in the range of 20 nm–50 μm. These nanostructures are described as rolled sheet(s) of graphene built from hexagonal arrangement of sp^2^-hybridized carbon atoms and are classified as single-walled and multi-walled carbon nanotubes (SWCNTs and MWCNTs), depending on the number of graphene layers (6,7).

Although CNTs have found enormous popularity in nanotechnology and generated extraordinary interest and expectations for biomedical applications. however, these new potential applications call for thorough studies on biocompatibility and toxicological burden. The identification of possible risks to human health is an essential prerequisite for a successful introduction of CNTs in future technological and biomedical applications. Pristine SWCNTs are chemically inert, extremely hydrophobic tubes and are therefore, insoluble in aqueous media. Despite the valuable potentials of CNTs in drug delivery, due to the hydrophobicity, tendency to aggregate, small size and extreme aspect ratio, they are harmful to living cells and hence, their biological and medical applications are then highly limited (8). Results obtained by several researchers have shown that raw, non-functionalized CNTs can potentially cause adverse effects, such that the general approach is to consider CNTs as toxic. Well-functionalization (either covalently or non-covalently) of CNTs with biocompatible surface coatings has been shown to reduce the toxic effects remarkably, while increasing their biocompatibility (the higher the degree of functionalization, the safer is the material). Functionalized, water soluble CNTs can also be well tolerated *in-vivo* and taken up by cells to a considerable degree in an energy-independent manner and therefore, have a specific capacity to cross cell membranes (9-15).

The aim of the main project was to use SWCNT-based nanostructures as efficient carriers for drug loading and delivery. As the first step, in our previous study, we functionalized SWCNTs non-covalently with two commercially available phospholipid polyethylene glycols (Pl-PEG 2000 and Pl-PEG 5000) to overcome the problems associated with the solubility of SWCNTs and evaluated the effect of PEGylation on their solubility (16). The results of the previous study showed that our non-covalent functionalization protocol could considerably increase aqueous solubility. As the second step, we hypothesized that through non-covalent functionalization with Pl-PEGs, the toxicity of the Pl-PEG-SWCNTs conjugates would be considerably reduced and therefore, the present investigation was planned to study the effect of SWCNTs PEGylation on viability and proliferation of Jurkat cell line (human tumor T lymphocytes) as a model for immune cells of lymphoid origin. 

## Experimentals

SWCNTs (P2-SWCNTs) with D_50_ of 4.03 nm, (carbonaceous purity of 90%, metal content of 4%–7%, prepared by chemical vaporization deposition technique) were purchased as the standards from Carbon Solutions Company (California, USA). Pl-PEG 5000-SWCNTs and Pl-PEG 2000-SWCNTs were prepared based on the method described previously (16). RPMI 1640, streptomycin, penicillin and fetal bovine serum (FBS) were purchased from Gibco Invitrogen Company (USA). Trypan blue was provided from Merck Company (Germany), dimethyl sulfoxide (DMSO) 99.7%, propidium iodide (PI) and DNase free RNase were purchased from Sigma Company (Germany). 

*C* *ell culture*

Jurkat E6.1 cell line with the following specifications was obtained from National Cell Bank of Iran (NCBI) and used in this study: ATCC Number: TIB-152. NCBI Code: C121.Designation: Jurkat. Morphology: Lymphoblast-like.Type: Non-adherent.

The cells were cultured in RPMI 1640 medium that were supplemented with 2 mM L-glutamine, 10% FBS, 100 IU/mL penicillin and 100 IU/mL streptomycin. The culture media were then kept in a controlled atmosphere (5% CO_2_) and incubated at 37°C (Astecc AV114C, UK). These experiments were performed on cells in the logarithmic phase of growth, in conditions of excellent viability (98%) as assessed by trypan blue exclusion method.

*Cytotoxicity assay*

Jurkat cells were first centrifuged at 1000 rpm for 10 min (Hettich D-78532, Germany). The cell pellets were washed in duplicate by resuspending in 5 mL normal saline. Following the final centrifugation, the cell pellets were resuspended in complete RPMI medium. Jurkat cells were then transferred into sterile falcons at the concentration of 1 × 10^6^ cells*/*mL. Various concentrations of test materials, including pure SWCNTs (final concentrations of 10, 50, 100, 150, 250 and 300 µg/mL), Pl-PEG 2000-SWCNTs and Pl-PEG 5000-SWCNTs (final concentrations of 10, 50, 100, 150, 250, 300, 350, 450, 550 µg/mL) were prepared in the culture medium. Jurkat cells were then incubated for 3 h at 37°C with test materials and seeded in 6-well culture plates at the concentration of 1 × 10^6^ cells*/*well. The plates were incubated for 24, 48 and 72 h at 37°C in a 5% CO_2_ humidified incubator (Astecc AV114C, UK). All experiments were performed in triplicate (14, 17-20).

*Cell viability and proliferation assay*

Trypan blue exclusion test was used to evaluate the effects of non-functionalized and functionalized SWCNTs on the viability of Jurkat cells. Cell proliferation and the number of viable cells were measured by counting the cells in a Neubauer hemocytometer after 24, 48 and 72 h exposure to pure SWCNTs, Pl-PEG 2000-SWCNTs and Pl-PEG 5000-SWCNTs.

*Analysis of apoptosis by flow cytometry*

Flow cytometry was used to study and analyze the cell cycle kinetic status of Jurkat cells, using PI staining method, as described previously (21). Jurkat cells were first centrifuged at 1000 rpm for 10 min. The cell pellets were washed in duplicate by resuspending in 5 mL normal saline. Following the final centrifugation, the cell pellets were resuspended in complete RPMI medium. Jurkat cells were then transferred into sterile falcons at the concentration of 1 ×10^6^ cells*/*mL. Various concentrations of SWCNTs, Pl-PEG 2000-SWCNTs and Pl-PEG 5000-SWCNTs at the final concentrations of 150µg/mL were prepared in the culture medium. Jurkat cells were then incubated for 3 h at 37°C with the test materials and seeded in 6-well culture plates at a concentration of 1 × 10^6^ cells*/*well. The plates were incubated for 48 h at 37°C in an atmosphere of 5% CO_2_ in air. Once fixed with 70% ethanol at 4°C for 1 h in dark conditions, the cells (1 × 10^6^/mL) were washed with phosphate-buffered saline (PBS) and resuspended with 250 μL of 50 μg/mL RNase in 2.5% sodium tricitrate buffer (pH 8.2). Incubation was continued at 37°C for 30 min. The cells were then stained with 250 μL of PI solution (50 μg/mL) for 20 min. The treated cells were centrifuged, resuspended with PBS and the final cell suspension was kept on ice until analysis. The number of cells in different phases of the cell cycle was analyzed using a FAC Scan Flow cytometer equipped with Cell-FIT software (Becton Dicknson Instruments, USA) at 488 nm excitation and a filter of 615 nm for PI detection with analysis ability of at least 50,000 cells per sample.

**Table 1:** IC50 values obtained following the exposure of Jurkat cells with various concentrations of pure SWCNTs, Pl-PEG 2000-SWCNTs and Pl-PEG 2000-SWCNTs.

	**IC**_50_ ** (µg/mL)**		
	**24 h**	**48 h**	**72 h**
**Pure SWCNTs**	300	150	100
**Pl-PEG 2000-SWCNTs**	400	250	225
**Pl-PEG 5000-SWCNTs**	>550	450	400

*Cell morphology observation (double dye staining)*

Cell morphology was examined using a fluorescence microscope (Motic AE31 Moticam Pro 2828, China) by staining the cells with DNA-binding fluorescent dyes, propidium iodide and acridine orange simultaneously. Jurkat cells were incubated for 48 h in a medium containing 150µg/mL pure SWCNTs in a CO_2_ (5%) humidified incubator. Following complete washing with PBS, the cells were stained with PI (50 µg/mL) and acridine orange (5 µg/mL) solutions. The images of the cells were finally were taken by a camera equipped microscope.

## Results

**Figure 1:**
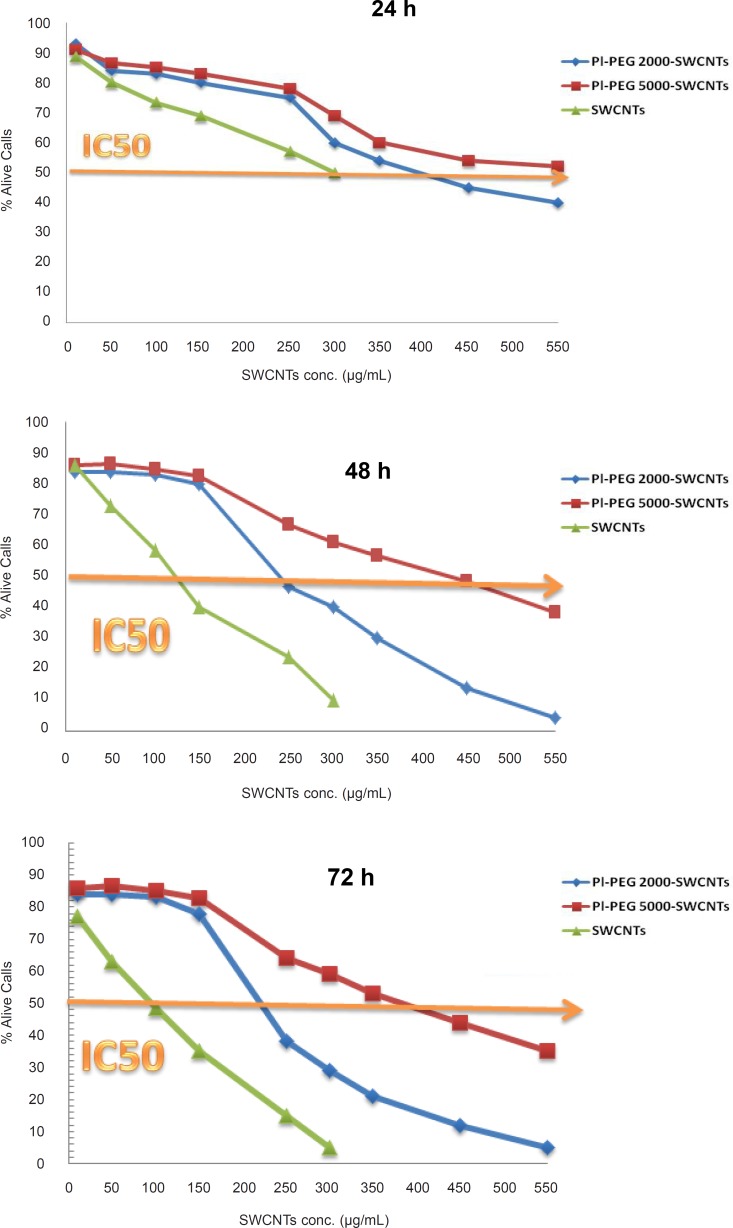
The effect of various concentrations of pure SWCNTs, Pl-PEG 2000-SWCNTs and Pl-PEG 5000-SWCNTs on the viability of Jurkat cells, following 24, 48 and 72 h (n = 3). Viability measured as (number of cells in each test group after 24 h÷ number of cells in control group after 24 h) × 100

In this study, Jurkat cells were treated for 24, 48, and 72 h with 10 to 300 µg/mL of pure SWCNTs and 10 to 550 µg/mL of functionalized SWCNTs (*f*-SWCNTs) and their effects on the cell viability were evaluated by trypan blue exclusion test. As depicted in Figure 1, all SWCNTs samples decreased the cell viability, the amount of which depended upon the time of exposure. However, both Pl-PEG-SWCNTs showed less toxic effects even at higher concentrations, compared with pristine CNTs. IC_50_ values in the presence of the investigated SWCNTs samples following various exposure times are presented in Table 1. Based on the results obtained, it seems that the inhibitory activity of pristine SWCNTs on Jurkat cells is considerably more than that of *f*-SWCNTs.

Both PEGylated SWCNTs caused a delayed phase in the occurrence of toxicity signs in Jurkat cells. Statistical analysis (one-way ANOVA) of data indicated a significant difference between the effects of PEGylated and non-PEGylated CNTs on the cell viability at the concentration range of no more than 150g/mL, regardless of the duration of treatment (p < 0.001). Nevertheless, it was observed that as the concentration of SWCNTs samples increased, a considerable difference between the toxicity profiles of PEGylated samples were also observed. Statistically, the significant difference appeared at the concentrations of 300 and 250 µg/mL for the 24-h and 48/72-h treatments, respectively (p < 0.001). These results suggest that not only functionalization with Pl-PEGs may alter the toxicity profile, but also the molecular weight of Pl-PEGs plays an important role at higher concentrations (the higher the molecular weight of the functionalizing group, the more improvement in biocompatibility of SWCNTs with Jurkat cells). 

**Figure 2:**
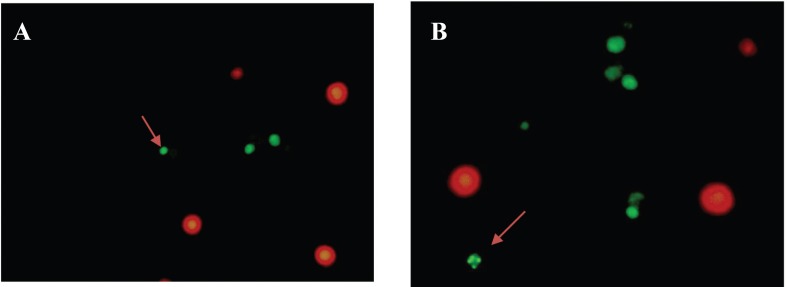
Florescence microscopy of Jurkat cells. Morphological changes of Jurkat cells as shown by the arrow for a: control group (cells not treated with pure SWCNTs), b: following the treatment with 150 μg/mL pure SWCNTs for 48 h

Microscopic examinations demonstrated that Jurkat cells grew and spread perfectly in the control group. Double staining method was used in order to distinguish apoptotic and necrotic cells. As seen in Figure 2A, the living cells are bright orange-colored, due to the fluorescence appearance of acridine orange bound on DNA. On the other hand, cells cultured with 150 µg/mL pure SWCNTs for 48 h exhibited featured characteristics of apoptosis such as cell body shrinkage, nuclear condensation and loss in cell membrane integrity (Figure 2B), whereas necrotic cells are red-colored due to the fluorescence appearance of PI bound on DNA. PI was used to assess the change in DNA and nuclear structure following exposure to SWCNTs. As depicted in Figure 2A, untreated Jurkat cells exhibited normal green and orange nuclei with organized cellular structures, whereas in the culture medium containing SWCNTs (Figure 2B), cells exhibited feature characteristics of apoptosis including membrane vesicles, nucleus condensation, fragmentation and apoptotic bodies. 

**Figure 3:**
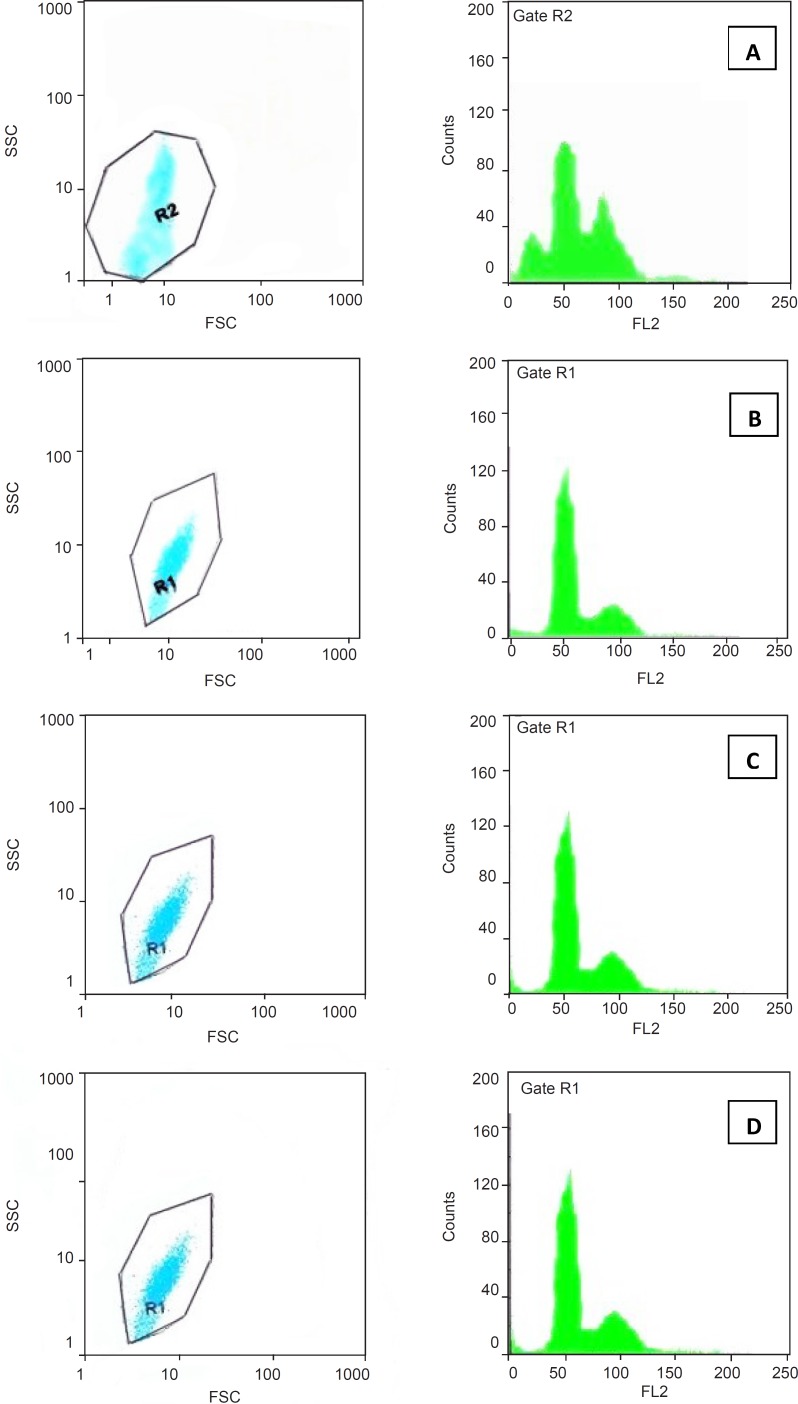
Cell cycle phase distribution of Jurkat cells cultured for 48 h with 150 μg/mL of a:pure SWCNTs, b: Pl-PEG 2000-SWCNTs, c: Pl-PEG 5000-SWCNTs in comparison with, d: control group, analyzed by flow cytometry.

In addition to the morphological evaluation, DNA content and cell-cycle phase distribution were analyzed by flow cytometry with PI staining. The results obtained by trypan blue staining suggested that pure SWCNTs were more toxic than *f*-SWCNTs at 48 h and hence, we used this exposure time for our following investigations on cell cycle and DNA content. Cell cycle phase distributions of Jurkat cells cultured with SWCNTs, Pl-PEG 2000-SWCNTs and Pl-PEG 5000-SWCNTs are shown in Table 2. As compared with the control, when the cells were exposed to 150 µg/mL pure SWCNTs and cultured for 48h the number of cells following the normal cell cycle of G_1_ and S phases continued to drop (Figure 3A). At the same time, the cell cycle was arrested in G_2_/M and 39.4% cells exhibited apoptotic feature. These results confirmed that SWCNTs could cause cell cycle arrest in G_2_/M and induce cell apoptosis which was detectable as sub G_1_ peak. However, Pl-PEG-SWCNTs seemed to have no similar effect at the same concentration (Figure 3B and 3C). Also, unlike SWCNTs, Pl-PEG-SWCNTs have not affected three phases of G_1_, S and G_2_/M, in comparison to the control group (Figure 3D), and the cells are following a normal cell cycle.

Plots of sideward and forward scatterings (SSC and FSC, respectively) are also shown in Figure 3. Light scattering, as measured by a flow cytometer, is a complex amalgam of the way particles reflect, refract, and diffract the light. The amount of scattered light depends upon several factors, notably cell size, nuclear/cytoplasmic ratio, granularity of the cytoplasm, surface topography, and the difference in the refractive index between the intra- and extracellular media. The light scattering properties of cells during death can change due to morphological changes such as cell swelling, cell shrinkage, rupture of plasma membrane, chromatin condensation, nucleus fragmentation and shedding of apoptotic bodies. Necrotic death is characterized by rapid initial increase in forward and sideward scattering due to cell swelling, whereas apoptotic death is characterized by a decrease in both forward and sideward scattering, although an initial increase in sideward scattering parallel with a decrease in forward scattering is seen in some cell types. In general, broken cells, isolated nuclei, cell debris and apoptotic bodies have low light scattering properties. Since light scattering analysis is specific to neither apoptosis nor necrosis, more mechanistic data can be obtained by combining this technique to another cytofluorometric analysis such as PI staining (22-24). In this study, we decided to combine light scattering changes with a cytofluorometric cytotoxicity assay of PI staining. As indicated in Figure 3, forward and sideward scatterings which are the parameters related to cell size and cell structure, respectively, are the same in two groups of cells exposed to Pl-PEG-SWCNTs (Figure 3B, 3C) and untreated cells (Figure 3D). However, a decrease in forward scattering and an increase in sideward scattering were observed for the cells treated with pure SWCNTs. This change in light scattering show that there are a number of apoptotic bodies in the SWCNTs treated cells are appeared as sub G_1_ peak in Figure 3A. 

**Table 2:** Cell cycle phase distribution of untreated and SWCNTs-treated (48 h) Jurkat cells.

	**% Distribution ratio**			
	**G**_1_	**S**	**G**_2_ **/M**	**Sub-G**_1_ **(Apoptotic cells)**
**SWCNTs (150 µg/mL)**	28.5 ± 2.1	39.8 ± 1.9	23.7 ± 1.1	39.4 ± 3.8
**Pl-PEG 2000-SWCNTs (150 µg/mL)**	37.8 ± 2.9	47.7 ± 3.3	12.5 ± 1.8	0.0
**Pl-PEG 5000-SWCNTs (150 µg/mL)**	37.4 ± 3.1	48.5 ± 2.8	13.1 ± 4.2	0.0
**Control (untreated)**	36.1 ± 1.7	49.1 ± 2.5	13.8 ± 2.8	0.0

Cytotoxicity studies and cell morphology investigation show that pure SWCNTs decreased the number of Jurkat cells in cultures, in a time-dose dependent manner. However, *f*-SWCNTs did not impair cells growth at higher doses in comparison to non-functionalized SWCNTs. The reduction of cell number could be a consequence of cell death. To prove this, we examined the process of cellular apoptosis by flow cytometry and double staining. Neither of examined *f*-SWCNTs was cytotoxic in doses below IC_50_ (150 µg/mL). However, SWCNTs induced apoptosis even at its IC_50_ concentration (150 µg/mL). The percentage of apoptotic cells was found to be around 39.4%, and therefore it can be declared that pure SWCNTs may cause toxic effects on Jurkat cells. However, PEGylation has been shown to be successful to alter the toxicity of SWCNTs on Jurkat cells. This was in agreement with the idea that proposed CNTs and CNTs without serum-stable functionalization show toxicity to cells at moderate dosage, while serum-stable, functionalized CNTs show little toxicity at higher doses (12).

## Discussion

Carbon nanotubes (CNTs) represent one of the most promising engineered nanomaterials, due to their extraordinary features which are exploited in many fields of technology and medicine with possible applications ranging from nanocomposites to new imaging systems or drug delivery technologies. However, the exceptional characteristics that make CNTs interesting for novel applications may also lead to possible hazardous effects on biological systems, particularly if they are used in medical fields (12).

It has been extensively mentioned in the literature that intrinsic poor solubility or dispersibility of CNTs in nearly all solvents and physiologic fluids is the most challenging obstacle. Despite the valuable potentials of CNTs in drug delivery, results obtained by several researchers have shown that raw, non-functionalized CNTs can potentially cause adverse effects, due to their small size and extreme aspect ratio, such that the general approach is to consider CNTs as toxic. A large number of studies have been performed to explore the potential toxic effects of CNTs (13, 25-37). The conclusions of all toxicity reports varied drastically, showing that the toxicity depends upon several factors, including structure, length, aspect ratio, surface area, degree of aggregation, method of preparation and surface topology. It has been postulated that by modifying the chemical nature of the surface, through functionalization of CNTs sidewalls either covalently or non-covalently, one can disperse CNTs in aqueous solvents uniformly and reduce the toxic effects remarkably while increasing their biocompatibility and in vivo tolerability (depending upon the degree of functionalization, type of bound functional groups, functionalization approaches, duration of exposure, method of exposure and the dispersant used to solubilize the nanotubes) (10, 12, 32, 38).

The cytotoxicity of pristine SWCNTs in human epidermal keratinocytes was investigated by Shvedova and his co-workers who demonstrated the formation of free radicals, accumulation of peroxidative products, depletion of total antioxidant reserves, and consequently a loss of cell viability (19, 25). Similar findings were obtained for T cell lines or primary human peripheral blood lymphocytes where pristine CNTs induced oxidative stress, plasma membrane depolarization or decreased metabolic activity (39). Results of comparative toxicity studies by Sayes and his co-workers indicated the highest toxicity with pristine CNTs and the lowest toxicity in the presence of *f*-CNTs, depending upon the density of functionalization (10, 19). Attachment of cationic ligands on the surface of CNTs was found to significantly improve the toxicity profile as a function of CNT surface cation density. Cationic *f*-CNTs are capable of entering both the non-adherent and adherent cell lines and have been reported to be nontoxic at concentrations below 0.05 mg/mL (40, 41). 

CNTs, without proper functionalization, may aggregate in the cell culture due to the highly hydrophobic surfaces and induce certain cell responses such as cell toxicity and death with apoptosis and necrosis being the most commonly observed mechanisms (19). Functionalization through the attachment of different functional groups can improve the solubility (dispersibility) and biocompatibility of CNTs. Polyethylene glycol (PEG) is increasingly used in biomedical and pharmaceutical applications, due to its biocompatibility and appropriate aqueous solubility. Functionalization of CNTs with PEG is a recognized good platform for the formulation of drug delivery systems. PEG-functionalized SWCNTs has been demonstrated to possess excellent stability without agglomeration in biological media (1, 12, 16, 20, 42-48). Heister and his colleagues declared that PEGylation resulted in a statistically significant enhancement in cell viability (83% compared to 75%) compared to RNA-wrapping approach of CNTs (44). They suggested that this effect might be due to either the better dispersion stability or the ability of PEGylation to reduce nonspecific uptake by cells. 

In first part of this study, a noncovalent functionalization method of SWCNTs by Pl-PEG 2000 and Pl-PEG 5000 was developed. Pl-PEG conjugates are amphiphilic compounds which contain two hydrophobic chains and hydrophilic head groups. The two hydrocarbon chains are able to strongly attach to the nanotube surface with the hydrophilic PEG chain extending into the aqueous phase, imparting desirable water solubility. We observed that our noncovalent functionalization protocol could considerably influence the aqueous solubility. As the next step, we decided to evaluate the toxicity effects of the Pl-PEG-SWCNTs conjugates on viability and proliferation of Jurkat cell line and answer this question whether or not the functionalization of SWCNTs through the PEGylation approach could decrease their cytotoxicity. Various methods for the cytotoxicity assay of SWCNTs are well described in the literature. Among the methods used, one can mention direct counting of cell numbers through trypan blue exclusion assay, colorimetric assay, measurement of protein concentrations, fluorescence-activated cell sorting flow cytometry and confocal microscopy assay (22, 37, 49). In the present research, trypan blue exclusion test was used to evaluate the effects of non-functionalized and functionalized SWCNTs on the viability of Jurkat cells, apoptosis was analyzed by flow cytometry and finally cell morphology was observed through double dye staining technique. In general, results obtained indicated that pristine SWCNTs exerted a dose-dependent toxicity and induced a relatively massive loss of Jurkat cell viability at doses above 150µg/mL, whereas PEGylated SWCNTs showed significantly less toxicity at higher concentrations, depending upon the type of Pl-PEG conjugates. 

## Conclusions

In spite of having a great potential for transport of drugs across the cell membrane into the cytoplasm and nucleus, the toxicity of CNTs needs to be evaluated. Careful in vitro studies would be very valuable to pre-screen nanomaterials for potential toxic effects on immune cells and provide additional insights on the mechanisms underlying the toxicity of these materials. Toward accomplishing this evaluation, we planned to expose Jurkat cells, as a model of immune cells, to pristine SWCNTs as well as PEGylated ones. Our observations showed that the non-covalent PEGylation protocol proposed in this project improved the SWCNTs biocompatibility significantly.
